# Genome sequences of five *Streptomyces* type strains (*Streptomyces argenteolus*, *Streptomyces pratensis*, *Streptomyces puniceus*, *Streptomyces spiroverticillatus*, and *Streptomyces sundarbansensis*)

**DOI:** 10.1128/mra.00308-26

**Published:** 2026-05-18

**Authors:** Adrien Biessy, Anuradha Jayathissa, William Jordan, Megan Bennett, Molly Calo, Martin Filion

**Affiliations:** 1 Department of Plant Science, McGill University, MacDonald Campus 151165, Sainte-Anne-de-Bellevue, Quebec, Canada; University of Strathclyde, Glasgow, United Kingdom

**Keywords:** genomes, *Streptomyces*

## Abstract

The genus *Streptomyces* is one of the largest prokaryotic taxa, including more than 800 species. Sequencing type strain genomes is essential to facilitate the identification of new isolates and the description of novel species. Here, we present the genome sequences of five *Streptomyces* type strains.

## ANNOUNCEMENT

The genus *Streptomyces* is one of the most important sources of antibiotics and therapeutic drugs (1, 2). It is also one of the largest prokaryotic genera, comprising more than 800 species with validly published and correct names (3, 4). Generating high-quality genomes for all type strains is critical, as it facilitates the identification of new isolates and the description of novel species within this large and important genus. In this context, we sequenced the genomes of five *Streptomyces* type strains (*Streptomyces argenteolus*, *Streptomyces pratensis*, *Streptomyces puniceus*, *Streptomyces spiroverticillatus*, and *Streptomyces sundarbansensis*) that belong to—or are closely related to—the *Streptomyces griseus* clade and for which high-quality genomes were previously unavailable. These type strains were isolated from diverse habitats, including grassy field soil and mangrove forest sediments (5, 6).

The strains were obtained from the Leibniz Institute DSMZ-German Collection of Microorganisms and Cell Cultures in freeze-dried form. They were revived in tryptic soy broth (BD Difco) at 28°C under agitation (150 rpm). Genomic DNA was extracted from 5-day-old cultures using the DNeasy PowerLyzer Microbial Kit (Qiagen) following the manufacturer’s instructions. DNA quality and concentration were checked using a NanoDrop ND-1000 UV/Vis spectrophotometer. Library preparation and genome sequencing were performed at the Integrated Microbiome Resource (Halifax, NS, Canada). Genomic DNA was enzymatically fragmented and indexed using the LongPlex Long Fragment Multiplexing Kit (seqWell) according to the manufacturer’s instructions. The resulting DNA fragments (~8–10 kb) were cleaned up and pooled before undergoing library preparation using the SMRTbell Prep Kit 3.0 (Pacific Biosciences) as per the manufacturer’s instructions. Genome sequencing was performed on a Vega sequencer (Pacific Biosciences) loaded with one Vega SMRT Cell (Vega chemistry). SMRT Link v25.3 (Pacific Biosciences) was used for demultiplexing and adaptor trimming. FastQC v0.11.9 (7) was used to check the quality of the HiFi reads. No read filtering or error correction was performed. Genome assembly was performed with Flye v2.9.5-b1801 using the command “--pacbio-hifi.” The genomes were annotated by the National Center for Biotechnology Information (NCBI) Prokaryotic Genome Annotation Pipeline v6.10 (8). Default parameters were used for all software unless otherwise specified. Sequencing, assembly metrics, and genome features are presented in Table 1.

**TABLE 1:** Genome sequencing/assembly metrics and genome features^*a*^

Data	Type strains				
	DSM 40036^T^	DSM 40083^T^	DSM 40226^T^	DSM 42019^T^	DSM 105114^T^
Species	*Streptomyces spiroverticillatus*	*Streptomyces puniceus* (syn. *Streptomyces californicus*)	*Streptomyces argenteolus*	*Streptomyces sundarbansensis*	*Streptomyces pratensis*
Isolation date	1955	Unknown	Unknown	2001	Unknown
Origin	Soil, Japan	Unknown	Soil	Mangrove forest sediments, India	Grassy field soil, Charlotte, NC, USA
No. of HiFi reads	27,080	732,127	106,945	92,162	249,226
HiFi read N50 (bp)	5,410	8,187	5,641	5,423	6,226
Coverage (x)	13	259	75	59	175
Genome size (bp)	9,797,833	8,221,749	7,128,956	7,479,561	7,690,231
G + C content (%)	71.2	72.5	71.0	72.0	71.0
No. of contigs	20	3	4	2	12
Contig N50 (bp)	1,259,728	7,919,793	6,741,712	7,232,247	7,544,179
No. of coding DNA sequences	8,608	7,149	6,250	6,445	6,728
No. of pseudogenes	163	100	182	133	126
No. of rRNAs	23	18	18	18	18
No. of tRNAs	69	72	68	67	68
Closest type strain from another species (accession no.)	*Streptomyces finlayi* JCM 4637^T^ (NZ_BMVC00000000.1)	*Streptomyces californicus* NRRL B-2098^T^ (CP070241.1- CP070243.1)	*Kitasatospora cinereorecta* JCM 6916^T^ (JBHUET000000000.1)	*Streptomyces rubiginosohelvolus* JCM 4415^T^ (BMTW00000000.1)	*Kitasatospora papulosa* NRRL B-16504^T^ (JNYQ00000000.1)
Digital DNA-DNA hybridization value (%)	99.8	87.5	96.5	50.6	94.8
GenBank accession no.	JBVQTE000000000	JBTRAP000000000	JBTRAO000000000	JBTRAN000000000	JBVQTD000000000
SRA accession no.	SRR36161494	SRR36161493	SRR36161492	SRR36161491	SRR36161490

^*a*^ The most closely related type strains were determined by TYGS, except for *S*. *argenteolus* DSM 40226^T^. For this strain, the most closely related type strain (*Kitasatospora cinereorecta* JCM 6916^T^) was identified by the taxonomy check performed by the NCBI.

**Fig 1:**
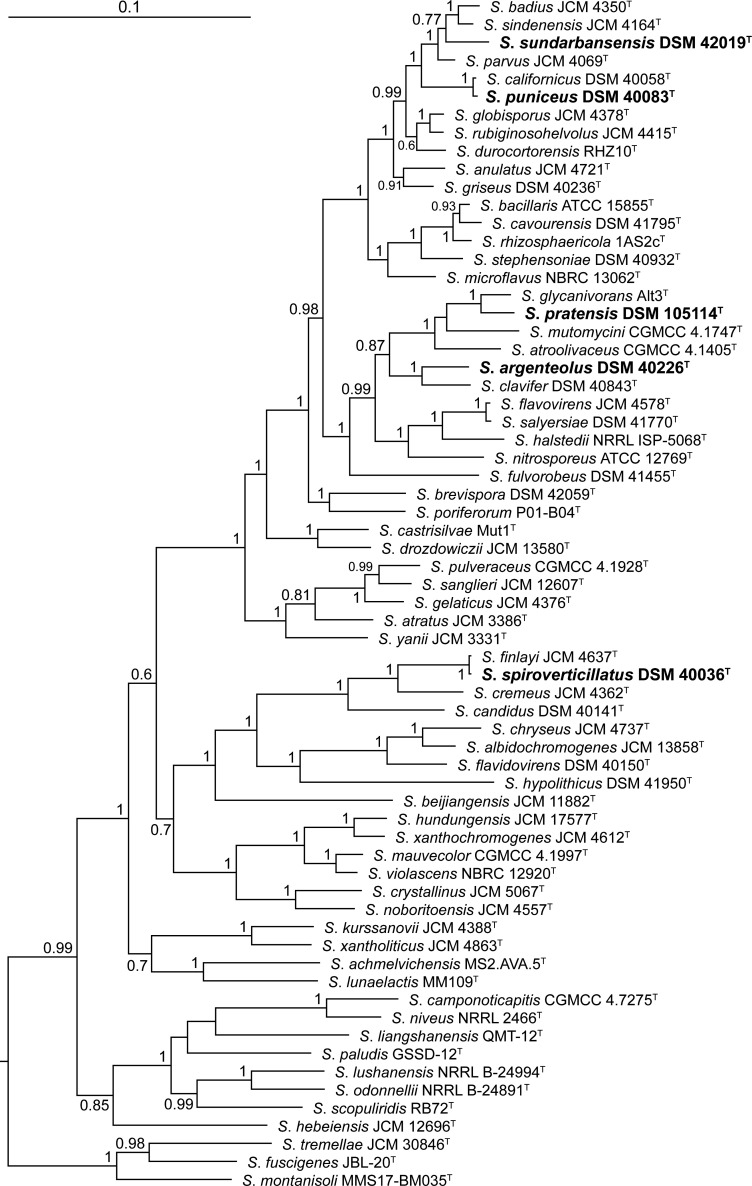
Phylogenetic relationships between the five *Streptomyces* type strains under study and various *Streptomyces* type strains. Genomes of closely related *Streptomyces* type strains were downloaded from GenBank. The complete nucleotide sequences of five housekeeping genes (*atpD*, *gyrB*, *recA*, *rpoB*, and *trpB*) were extracted from each genome, aligned using Clustal Omega v1.2.2 (13), and subsequently concatenated. The resulting alignment was used to generate a phylogenetic tree using FastTree v2.1 (14) and the generalized time reversible model. The tree topology was verified by computing Shimodaira Hasegawa support values (15). Only support values above 0.5 are displayed at the nodes. *Actinomyces bovis* NCTC11535 ^T^ was used as an outgroup (not shown on the tree). The five strains whose genomes were sequenced in this study are highlighted in bold. The scale bar indicates sequence divergence.

The five genomes were assembled into 2–20 linear contigs, with total genome sizes ranging from 7.13 to 9.80 Mb and G + C content varying from 71.0% to 72.5%. To visualize the phylogenetic relationships of the five type strains with other *Streptomyces* species, a multilocus sequence analysis was performed (Fig. 1). Digital DNA-DNA hybridization (dDDH) values between the five type strains under study and closely related type strains were also calculated using the Type (Strain) Genome Server v408 (9, 10) and the Genome-to-Genome Distance Calculator software v4.0 (11). According to the dDDH values presented in Table 1, *S. puniceus* is a later heterotypic synonym of *Streptomyces californicus*, as previously suggested (12). *S. spiroverticillatus*, *S. argenteolus*, and *S. pratensis* are earlier heterotypic synonyms of *Streptomyces finlayi*, *Kitasatospora cinereorecta*, and *Kitasatospora papulosa*, respectively.

## Data Availability

The genome sequences of the five *Streptomyces* type strains (BioProject PRJNA1368964) have been deposited in GenBank. The versions described in this paper are the first versions. The raw sequencing data have been deposited in the Sequence Read Archive. Accession numbers are provided in Table 1.
